# Vegetarian Diet Was Associated With a Lower Risk of Chronic Kidney Disease in Diabetic Patients

**DOI:** 10.3389/fnut.2022.843357

**Published:** 2022-04-26

**Authors:** Yi-Chou Hou, Hui-Fen Huang, Wen-Hsin Tsai, Sin-Yi Huang, Hao-Wen Liu, Jia-Sin Liu, Ko-Lin Kuo

**Affiliations:** ^1^Department of Internal Medicine, Cardinal Tien Hospital, New Taipei City, Taiwan; ^2^School of Medicine, Fu Jen Catholic University, New Taipei City, Taiwan; ^3^Graduate Institute of Clinical Medicine, College of Medicine, Taipei Medical University, Taipei, Taiwan; ^4^Department of Chinese Medicine, Taipei Tzu Chi Hospital, The Buddhist Tzu Chi Medical Foundation, New Taipei City, Taiwan; ^5^School of Post-Baccalaureate Chinese Medicine, Tzu Chi University, Hualien, Taiwan; ^6^Department of Pediatrics, Taipei Tzu Chi Hospital, Buddhist Tzu Chi Medical Foundation, Hualien, Taiwan; ^7^School of Medicine, Tzu Chi University, Hualien, Taiwan; ^8^Tai-Yang Otorhinolaryngology Clinic, New Taipei City, Taiwan; ^9^Division of Nephrology, Taipei Tzu Chi Hospital, Buddhist Tzu Chi Medical Foundation, New Taipei City, Taiwan

**Keywords:** diabetes mellitus, chronic kidney disease, vegan diet, lacto-ovo vegetarian diet, obesity, hyperuicemia

## Abstract

**Introduction:**

Diabetes mellitus (DM) is a pathological hyperglycemic state related to the dysregulation of insulin. Chronic kidney disease (CKD) is a common chronic complication in diabetic patients. A vegetarian diet could be one of the preventive strategies for the occurrence of CKD in patients with diabetes mellitus. However, it is still unknown whether a vegetarian diet lowers the occurrence of CKD in DM patients.

**Research Design and Methods:**

This retrospective study was conducted at Taipei Tzu Chi Hospital from 5 September 2005 to 31 December 2016. Subjects with an HbA1c level > 6.5% or previous history of diabetes mellitus elder than 40 years were grouped based on self-reported dietary habits (vegetarians, lacto-ovo vegetarians and omnivores) in the structured questionnaire. Structural equation modeling (SEM) was applied to estimate the direct and indirect effects of variables on the occurrence of chronic kidney disease.

**Results:**

Among these 2,797 subjects, the participants were grouped into dietary habits as vegans (*n* = 207), lacto-ovo vegetarians (*n* = 941) and omnivores (*n* = 1,649). The incidence of overall CKD was higher in the omnivore group [36.6% vs 30.4% (vegans) and 28.5% (lacto-ovo vegetarian), *p* < 0.001]. In the SEM model, after adjusting for age and sex, the lacto-ovo vegetarian [OR: 0.68, 95% confidence interval (CI): 0.57–0.82] and vegan groups (OR 0.68, 95% CI: 0.49–0.94) were both associated with a lower risk of CKD occurrence than the omnivore group. The vegan diet and lacto-ovo diet lowered the risk related to a high BMI (OR: 0.45, *p* < 0.001, OR: 0.58, *p* < 0.001) and hyperuricemia (OR: 0.53, *p* < 0.001; OR: 0.55, *p* < 0.001) for the occurrence of CKD.

**Conclusion:**

Vegetarian dietary habits were associated with a lower occurrence of CKD in DM patients.

## Introduction

Diabetes mellitus (DM) is the pathological hyperglycemic state induced by insulin deficiency or resistance. A chronic hyperglycemic status could contribute to multiple organ dysfunction, including cardiovascular disease, peripheral neuropathy, retinopathy and nephropathy (1). The complications of diabetes mellitus influence patient survival and pose an economic burden for health expenditures; therefore, pharmacologic intervention and lifestyle modifications are important for controlling diabetes mellitus and its complications (2). Beyond pharmacologic strategies such as insulin or oral hyperglycemic agents, lifestyle behavior changes play an important adjunctive role in controlling hyperglycemic status. The Diabetes Prevention Program involving body weight loss and maintaining weekly physical activities has been advocated as the cornerstone for managing diabetic control (2018;^3^). Dietary counseling also plays an important role in preventing the development of DM. An adequate reduction in calories and fat helps lower the incidence of DM (4), and specific eating habits, such as the Mediterranean-style, Dietary Approaches to Stop Hypertension (DASH) or plant-based diet, are important for the prevention of DM (3, 5,6).

Chronic kidney disease (CKD) is characterized by a progressive decline in glomerular filtration rate or persistent proteinuria for more than 3 months (7). Diabetes mellitus, either type 1 or type 2, is a major metabolic etiology that contributes to CKD (8). At the same time, CKD itself disturbs insulin sensitivity by hyperactivity of the sympathetic tone, renin-angiotensin-aldosterone system and chronic inflammation (9–12). Hyperglycemic status enhances the hyperfiltration of the glomerulus and therefore worsens glomerular hypertrophy and sequential glomerular fibrosis (12). To lessen glomerular hypertrophy and downstream glomerular fibrosis, protein restriction is the main dietary intervention (13). As mentioned in the previous section, a Mediterranean-style diet or plant-based diet is suggested because the reduced protein content might provide benefits in relieving glomerular hypertrophy (14,15).

A vegetarian diet is one strategy for lowering protein ingestion. A vegetarian diet, which is composed of plant-based food, involves the consumption of grains, fruit, vegetables and unsaturated fat. Fish, meat and poultry products are excluded. In the lacto-ovo vegetarian diet, milk, dairy products and eggs are included. In the vegetarian diet, soy, wheat and nuts serve as the major sources of protein without an excessive reduction in calories. Previous studies indicate that a vegetarian-based diet is safe for CKD patients (16), and it plays several protective roles in delaying the initiation of renal replacement therapy (17). Previous cohort studies also provided evidence that a vegetarian diet influenced blood pressure control in CKD patients. Liu et al. demonstrated that a lacto-ovo dietary habit was associated with better blood pressure control in patients with proteinuria (18). Lacto-ovo vegetarian habits also provided better phosphate and lipid control in moderate CKD patients (19).

Based on the evidence above, a vegetarian diet might provide a protective role in CKD patients when protein restriction is the cornerstone of daily care. From our previous study, the vegan and lacto-ovo vegetarian habits provided a protective role in lowering the incidence of CKD (20). However, the role of vegetarian dietary habits in protecting against the occurrence of CKD in DM patients is unknown. The aim of the study was to investigate whether healthy dietary habits, especially vegetarian-based diets, are associated with the occurrence of chronic kidney disease in DM patients.

## Research Design and Method

### Study Participants

This retrospective study was conducted at Taipei Tzu Chi Hospital from 5 September 2005 to 31 December 2016 in Taiwan. The database was composed of individuals receiving self-paid health exams at the health checkup center in Taipei Tzu Chi Hospital (New Taipei City, Taiwan). The inclusion criteria were (1) subjects older than 40 years old and (2) subjects with serum hemoglobin A1c (HbA1c) levels > 6.5% or previous history of diabetes mellitus reported by the subjects. The exclusion criteria included participants without correct identification numbers or insufficient biochemical data. The study was approved by the institutional board of Taipei Tzu-Chi Hospital based on the Declaration of Helsinki (06-XD12-033). Further written informed consent were waived in this retrospective study by the ethical committee of Taipei Tzu-Chi Hospital.

### Clinical Assessment

We used the structured questionnaire applied in the studies by Chiu et al. except the food questionnaire (21) from Tzu-Chi medical system. After enrollment, a comprehensive health examination would be performed. Trained research nurse interviewed the participants with the questionnaire with gender, medical history, age, lifestyle habits (including smoking, alcohol and physical activities) and dietary habit. The subjects were grouped based on self-reported dietary habits: vegans, lacto-ovo vegetarian and omnivore. The lacto-ovo vegetarian was defined as an individual who consumed eggs or dairy products or both but no other animal products; a vegan was defined as one who consumed only plant-based foods; an omnivore was defined as one who consumed both plant- and animal-based foods.

An automatic electronic meter (SECA GM-1000, Seoul, South Korea) was used to measure height and weight. The body mass index (BMI, kg/m^2^) was calculated based on the measured body weight and height by a well-trained nurse. Blood pressure was measured by an automatic blood pressure machine (Welch Allyn 53000, NJ, United States).

Venous blood was drawn after patients had fasted for at least 12 h. Measurements included levels of serum uric acid, total cholesterol (TCH), triglycerides (TG), and high-density lipoprotein cholesterol (HDL-C) (Dimension RXL Max integrated chemistry system, Siemens, Erlangen, Germany). Serum creatinine was measured using the alkaline picrate (Jaffe) method. The estimated glomerular filtration rate calculation was based on the Chronic Kidney Disease Epidemiology Collaboration (CKD-EPI) from serum creatinine (22). Hyperuricemia was defined if the serum uric acid level was higher than 7 mg/dL in males and 6 mg/dL in females (23).

Urine protein was determined by an automated urine analyzer (Arkray 4030, Tokyo, Japan) analyzing a single dipstick. The severity of proteinuria was graded into six categories: absent (less than 10 mg/dL), trace (±) (10 to 20 mg/dL), 1 + (30 mg/dL), 2 + (100 mg/dL), 3 + (300 mg/dL) or 4 + (1,000 mg/dL). Patients with trace levels, 1 + level and above were defined as having proteinuria. The presence of CKD was defined as either the presence of proteinuria or an estimated glomerular filtration rate (eGFR) ≤ 60 mL/min per 1.73 m^2^ (7).

### Statistics

To compare the normal and continuous variables between the three groups (vegan, ovo-lacto vegetarian, or omnivore), chi-square and one-way ANOVA were applied. When there were fewer than 5 observed values or the data did not conform to a categorical distribution, Fisher’s exact test and the Kruskal–Wallis test were used instead. A multivariable logistic model was used to calculate the adjusted odds ratio (OR). Four separate logistic regression models were applied: an unadjusted model; a crude model (Model 1); a model adjusted for age and gender (Model 2); and a model adjusted for all the other parameters [full model (Model 3)]. The stepwise backward and likelihood ratio test were chosen as the approach for model selection.

### Structural Equation Modeling Model

Structural equation modeling (SEM) with Bernoulli distribution in a logistic regression model estimated the direct and indirect effects of the vegetarian, lacto-ovo vegetarian and omnivore diets and other research factors on CKD risk in diabetic patients. We assessed the relationships at two levels, including (1) the direct effects of CKD risk factors on CKD and (2) the indirect effects of vegetarian, lacto-ovo vegetarian and omnivore diets on CKD risk factors. We showed the adjusted odds ratios and 95% confidence intervals and *p* values.

### Variables Assessed in Structural Equation Modeling

We estimated the association with several factors and CKD. In addition, the model also assessed the biochemical values and relative index mediated by the vegetarian and lacto-ovo vegetarian diets compared to the omnivore diet in our SEM model. The biochemical values and relative indices were SBP, HbA1c level, BMI greater than 27, TG over HDL ratio, and high uric acid level. The two-tailed test was used for statistical significance testing, and a *p*-value < 0.05 was considered significant. When we used the Bonferroni adjustment to assess the difference between the variables, the value was still less than 0.05 for vegetarian versus both subgroups. The study assumes that the statistical significance level was 95% and power was 80%, and the proportion of CKD in diabetic patients was 40%. If the odds ratio of vegetarians to CKD was 0.8, the required sample was 2,774. We assessed the adequacy of the sample size based on the above calculations. All statistical analyses were executed with SAS software version 9.4 (SAS Institute, Inc., Cary, NC, United States) and STATA15.1 (Stata Corp, College Station, TX, United States).

## Results

**FIGURE 1:**
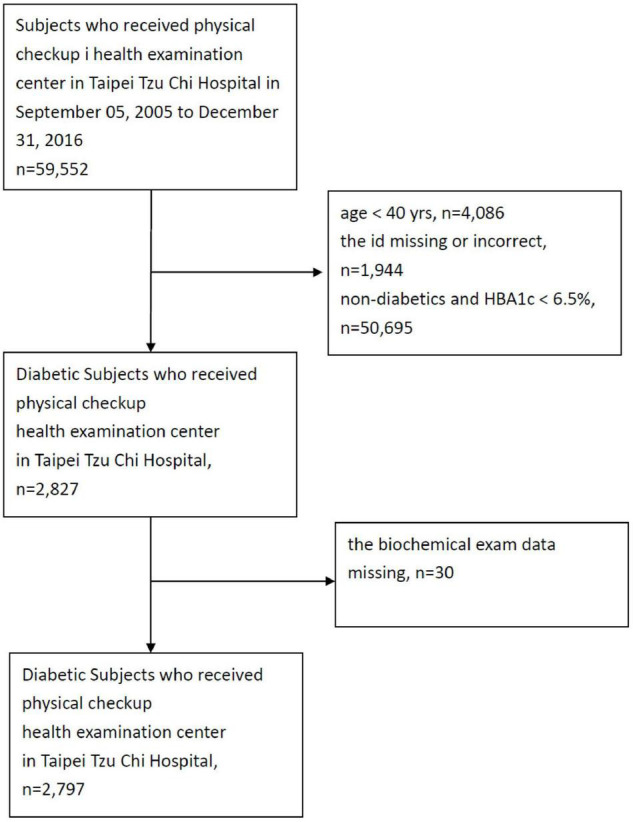
The flow chart for subject selection process in this study.

Figure 1 illustrates the flow chart of the enrollment within the study. The total database included 55,929 individuals. After the first exclusion for individuals younger than 40 years old (*n* = 4,086), individuals with incomplete identification (*n* = 1,944), individuals with HbA1c levels < 6.5% (*n* = 50,695) and individuals with incomplete or missing biochemical exam results (*n* = 30), the total number of subjects with DM within the cohort was 2,797. Among these 2,797 subjects, the participants were grouped by dietary habits as vegans (*n* = 207), lacto-ovo vegetarians (*n* = 941) and omnivores (*n* = 1,649).

### Demographic Information of the Subjects With Different Eating Habits

Table 1 displays the demographic information between the groups. There were 207 and 941 participants with vegan and lacto-ovo vegetarian diets, respectively, both of which were lower than the number of participants with an omnivore diet (*n* = 1,649, *p* < 0.001). The age of the participants with an omnivore diet (61.8 ± 10.7 years old) was lower than that of the participants with a vegan or lacto-ovo vegetarian diet (67.5 ± 8.9 and 64.3 ± 8.4 years old, respectively, *p* < 0.001). Females were less common in the omnivore group [38.7 vs 60.4% (vegans) and 60.4% (lacto-ovo vegetarian), *p* < 0.001]. Chronic exposure to cigarettes was more prevalent in the omnivore group [12.6 vs 1.9% (vegans) and 1.1% (lacto-ovo vegetarian), *p* < 0.001]. Among the physiological parameters, the omnivore group had a higher BMI than the other groups [25.5 ± 4.1 (kg/m^2^) vs 24.4 ± 3.8 kg/m^2^ (vegans) and 24.3 ± 3.7 kg/m^2^, (lacto-ovo vegetarians) *p* < 0.001]. The percentage of patients with hypertension was higher in the omnivore group [38.6 vs 33.3% (lacto-ovo vegetarian) and 32.9% (vegans), *p* = 0.002]. Among the biochemical parameters, HbA1c level (7.2 ± 1.6%, *p* < 0.001), the ratio of triglyceride/high-density lipoprotein (5.7 ± 1.6, *p* < 0.001) and the percentage of participants with hyperuricemia (14.8%, *p* < 0.001) were all higher in the omnivore group. Regarding the parameters indicating CKD, the omnivore group had a higher incidence of proteinuria [27.7 vs 21.7% (vegans) and 20.5% (lacto-ovo vegetarian), *p* < 0.001]. The proportion of participants with stage 1-2 CKD was also higher in the omnivore group [21.4 vs 14.5% (vegans) and 16.6% (lacto-ovo vegetarian), *p* = 0.002]. The incidence of overall CKD was higher in the omnivore group [36.6 vs 30.4% (vegans) and 28.5% (lacto-ovo vegetarian), *p* < 0.001].

**TABLE 1:** The demographic characteristic of subjects in the community.

	Vegan	Lacto-ovo vegetarians	Omnivore diet	*p*-value
*N*	207	941	1,649	<0.001
**Age group, years-old, *n* (%)**				
40–49	5 (2.4)	26 (2.8)	109 (6.6)	<0.001
50–69	14 (6.8)	150 (15.9)	376 (22.8)	<0.001
60–69	77 (37.2)	386 (41.0)	583 (35.4)	0.016
>70	111 (53.6)	375 (39.9)	540 (32.7)	<0.001
Age, years-old, mean(SD)	67.5 (8.9)	64.3 (8.4)	61.8 (10.7)	<0.001
**Gender, *n* (%)**				
male	82 (39.6)	373 (39.6)	1011 (61.3)	<0.001
female	125 (60.4)	568 (60.4)	638 (38.7)	<0.001
Current smoking, n (%)	4 (1.9)	10 (1.1)	208 (12.6)	<0.001
BMI, Kg/m^2,^ mean(SD)	24.4 (3.8)	24.3 (3.7)	25.5 (4.1)	<0.001
> 27, n (%)	41 (19.8)	196 (20.8)	526 (31.9)	<0.001
Systolic BP, mmHg, mean(SD)	126 (17)	125 (16)	126 (15)	0.42
Hypertension, n (%)	68 (32.9)	313 (33.3)	637 (38.6)	0.013
HbA1c,%, mean(SD)	7.1 (1.9)	6.8 (1.5)	7.2 (1.6)	0.002
TG/HDL ratio, mean(SD)	3.5 (3.2)	3.3 (3.1)	4 (4.2)	<0.001
Uric acid, mg/dL, mean(SD)	5.2 (1.3)	5.3 (1.4)	5.7 (1.6)	<0.001
Hyperuricemia, n (%)	15 (7.2)	77 (8.2)	244 (14.8)	<0.001
Proteinuria, n (%)	45 (21.7)	193 (20.5)	457 (27.7)	<0.001
Creatinine, mg/dL, mean(SD)	0.9 (0.7)	0.9 (0.4)	1.0 (0.4)	<0.001
CKD-EPI eGFR, mL/min/1.73 m^2^, mean(SD)	76 (16)	79 (15)	78 (17)	0.99
**CKD stage, n (%)**				
1–2	30 (14.5)	156 (16.6)	353 (21.4)	0.002
3	31 (15.0)	108 (11.5)	233 (14.1)	0.12
4–5	2 (1.0)	4 (0.4)	17 (1.0)	0.25
CKD, n (%)	63 (30.4)	268 (28.5)	603 (36.6)	<0.001

*SD: standard deviation.*

*BMI: body mass index, Systolic BP: systolic blood pressure, HbA1c: hemoglobin A1c.*

*TG/HDL ratio: triglyceride to high-density lipoprotein cholesterol ratio.*

*CKD, chronic kidney disease.*

### The Odds Ratio for the Occurrence of Chronic Kidney Disease by Demographic Factors and the Different Dietary Habits in DM Individuals in the Logistic Regression Model

Table 2 displays the odds ratio for CKD by risk factors such as physiological and biochemical parameters and dietary habits. In the crude logistic regression model, older age (OR 1.03, 95% CI: 1.02–1.04), male sex (OR 1.41, 95% CI: 1.21–1.67), smoking habit (OR 1.51, 95% CI: 1.15–2.00), BMI > 27 kg/m^2^ (OR 1.58, 95% CI: 1.33–1.88), every 10 mmHg increase in systolic pressure (OR 1.18, 95% CI: 1.12–1.24), HbA1c level (OR 1.15, 95% CI: 1.10–1.21), the ratio of triglycerides to high-density lipoprotein (OR 1.05, 95% CI: 1.02–1.07) and the occurrence of hyperuricemia (OR 2.00, 95% CI: 1.60–2.53) all posed hazards for the occurrence of CKD in diabetic patients. When compared with the omnivore diet, the lacto-ovo vegetarian diet had a protective effect against the occurrence of CKD (OR 69, 95% CI: 0.58–0.82). The vegan diet also had a lower risk of CKD (vs omnivores, OR 0.76, 95% CI: 0.56–1.04). When adjusting for age and sex, lacto-ovo vegetarian (OR: 0.68, 95% CI: 0.57–0.82) and vegan habits (OR 0.68, 95% CI: 0.49–0.94) may both have a lower risk of CKD. In the full model adjustment, lacto-ovo vegetarians showed a lower risk of CKD occurrence (OR 0.78, 95% CI: 0.65–0.95).

**TABLE 2:** Risk in different demographic characteristics and eating habits for occurrence of CKD (*n* = 2,797).

	Model 1 Odd ratio (95% confidence interval)	Model 2 Odd ratio (95% confidence interval)	Model 3 Odd ratio (95% confidence interval)
Vegan vs. Omnivores	0.76 (0.56–1.04)	0.68 (0.49–0.94)	0.75 (0.54−1.04)
Lacto-ovo vegetarian vs. Omnivores	0.69 (0.58–0.82)	0.68 (0.57–0.82)	0.78 (0.65−0.95)
Age per years old	1.03 (1.02–1.04)	1.03 (1.02–1.04)	1.03 (1.02−1.04)
Male vs. female	1.42 (1.21–1.67)	1.38 (1.17–1.62)	1.24 (1.04−1.47)
Current smoking	1.51 (1.15–2.00)		1.40 (1.03−1.90)
BMI > 27	1.58 (1.33–1.88)		1.32 (1.09−1.59)
Systolic BP, per 10 mmHg	1.18 (1.12–1.24)		1.09 (1.04−1.16)
HbA1c	1.15 (1.10–1.21)		1.12 (1.07−1.18)
TG/HDL ratio	1.05 (1.02–1.07)		1.03 (1.01−1.05)
Hyperuricemia	2.00 (1.60–2.53)		1.80 (1.42−2.29)

*MI: body mass index, Systolic BP: systolic blood pressure, HbA1c: hemoglobin A1c.*

*TG/HDL ratio: triglyceride to high-density lipoprotein cholesterol ratio.*

*CKD, chronic kidney disease.*

*Model 1 crude model.*

*Model 2 adjusted age, gender, vegan, lacto-ovo vegetarians and omnivores.*

*Model 3 adjusted the variables in model 2 and current smoking, BMI > 27, systolic BP, HbA1c, TG/HDL ratio and hyperuricemia.*

### The Adjunctively Lowering Effect of Chronic Kidney Disease Occurrence by Different Dietary Habits in DM Individuals in the Structural Equation Modeling Model

**FIGURE 2:**
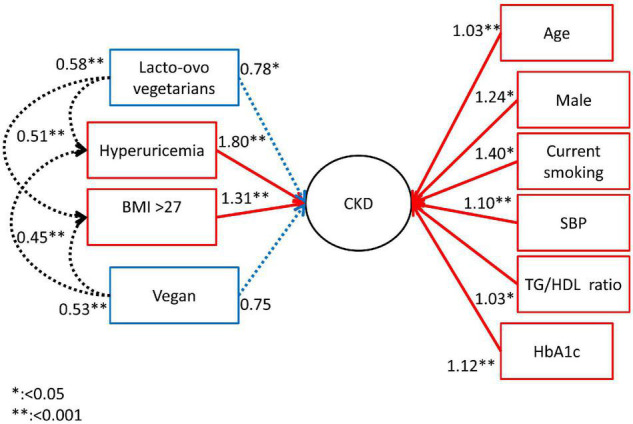
The odds ratio of risk factors on CKD in SEM model.

Figure 2 shows the effect of dietary habits on the interactions of the risk factors for CKD in DM patients by using the SEM model. The lacto-ovo diet had the direct effect on lowering the occurrence of CKD. The vegan diet did not lower the occurrence of CKD in SEM model, although it provided the possible protective effect after adjusting age and gender in model 2. However, a vegan diet lowered the risk related to higher BMI (OR: 0.45, *p* < 0.001) and hyperuricemia (OR: 0.53, *p* = 0.004) for the occurrence of CKD. The lacto-ovo diet lowered the risk of CKD directly, as illustrated in Table 2. The Lacto-ovo diet also mitigated the risk related to higher BMI (OR: 0.58, *p* < 0.001) and hyperuricemia (OR: 0.55, *p* < 0.001) for the occurrence of CKD. It also meaning that the lacto-ovo-vegetarian diet also indirectly affects occurrence of CKD through effects on hyperuricemia acid and risk related to higher BMI.

## Discussion

We found that traditional risk factors, such as hypertension, obesity, hyperuricemia and consumption of cigarettes, were associated with the occurrence of CKD in DM patients and that vegetarian dietary habits were associated with a lower risk of hyperuricemia and BMI > 27 kg/m^2^ in the occurrence of CKD in the SEM model. Among the different dietary patterns, vegetarians and lacto-ovo vegetarians had a lower incidence of CKD than the omnivores. While the traditional risk factors pose hazards for the occurrence of CKD, lacto-ovo and vegetarian diets provided protective effects after adjusting for sex and age and other traditional risk factors in a multivariable logistic regression model.

Dietary intervention has been applied to alleviate the complications of metabolic diseases. In controlling hypertension, a dietary approach to stop hypertension encourages reduced ingestion of sodium and high consumption of whole grains and low-fat dairy products. During the past 2 decades, the dietary behavior trend changed in Taiwan. The concept of a healthy diet encouraged adults to increase the ingestion of vegetables, grains, and soy products and avoid excessive ingestion of red meat or animal oils (24). Vegan and lacto-ovo vegetarian diets also provide similar effects for controlling blood pressure. The major components of the vegetarian diet are nuts, wheat and soy-based protein. As described in the previous sections, protein restriction is the cornerstone of dietary intervention in treating CKD, and the proportion of protein might shift from meat to whole grains, legumes or soy-based food (25). In advanced CKD patients, the enhanced consumption of grains might accompany hyperphosphatemia, but the calories from grains could trade off energy and reduce total protein ingestion (25, 26). Whole grain food also improved blood sugar control in DM (26), which might reflect less severe CKD in DM patients (27). A clinical trial by Dobre et al. showed that 12 weeks of supplementation with β-glucan from the grains also lowered the production of trimethylamine N-oxide within the body (28). Soy-based foods are common in Eastern Asian countries, and a soy-based diet habit was associated with a lower incidence of mild cognitive impairment (29). A soy-based diet also improved the survival associated with cognitive impairment (30). The soy-base protein content also has a renoprotective effect. From the *in vivo* study by Chen et al. soy β-conglycinin could directly enhance insulin sensitivity and alleviate the activation of renin-angiotensin-aldosteronism in streptozotocin-treated Wistar rats. The histologic progression of DM nephropathy could be retarded after the administration of soy β-conglycinin (31). The increased expression of nephrin in streptozotocin-treated rats was noted if soy β-conglycinin was given in the diet (32). From previous *in vivo* studies, energy expenditure and energy gain increased in rats receiving a low-protein diet compared with rats receiving a normoproteic diet. At the same time, brown adipose tissue could be lessened by increasing insulin sensitivity even when sympathetic tone increased (33). Sympathetic hyperactivity is common in DM patients, and sympathetic hyperactivity is associated with higher cardiovascular comorbidity. Since a vegan diet provides benefits for insulin sensitivity and metabolic adjustment related to adipose tissue, our result is also consistent with the conclusions from other studies. In the multivariates logistic regression, the vegan diet, in comparison with lacto-ovo vegetarians diet, did not provide the protective effect in CKD in crude model. However, the protective effect was demonstrated after adjusting age and gender. The demographic result illustrated that the age in vegan group was higher than other groups, and the advanced age was a risk factor for development of CKD. Our result might illustrate that both lacto-ovo vegetarians and vegan diet might provide a protective role in decreasing the development of CKD in DM subjects.

The role of a vegan diet in alleviating CKD progression has aroused growing attention. Dietary intervention for CKD prevention or progression includes restriction of daily protein, salt and inorganic phosphorus (15). From the aspect of protein restriction, daily protein ingestion is an important strategy for lowering intraglomerular hypertension and reducing the generation of urea and acid accumulation within the body (13). A protein restriction strategy reduces the decline in glomerular filtration rate in CKD patients and therefore delays entry into dialysis. In the daily diet, processed food and meat have been regarded as sources of exogenous acid in CKD because of excessive catabolism. In addition, animal-based proteins are the major source of purine, which is converted to uric acid. Hyperuricemia is an important risk factor for cardiovascular comorbidities in patients with metabolic syndrome since it serves as an important source of inflammation and oxidative stress. The dietary approach to stop hypertension, which is composed of grains, fish, and milk rather than red meat, might contribute a partial effect in lowering serum uric acid. From the study by Miller et al. the DASH diet lowered the serum uric acid level compared with an omnivore diet (34). When comparing the vegetarian diet with the omnivore diet, the urate-lowering effects differed in different studies. The EPIC-Oxford study enrolled 65,429 subjects in the United Kingdom, and the results demonstrated that subjects fed vegetarian diets had higher serum concentrations of uric acid (35). From a study by Chiu et al. vegans had lower uric acid levels than non-vegetarians in the cohort study initiated in the Buddhist hospital in Taiwan (36). In a study by Chiu et al. a vegetarian diet lowered serum uric acid levels, and the lowering effect was more obvious in patients with hyperlipidemia and diuretic users. The urate-lowering effect was not observed in the DM patients from the cohort study by Chiu et al. but diuretics are commonly used in CKD patients for adequate control of body fluid and blood pressure. However, diuresis accompanies the enhanced reabsorption of urate from the proximal tubules. Therefore, a vegetarian diet might be an important intervention to manage hyperuricemia in patients with DM nephropathy.

The safety of the low-protein diet has been confirmed in multiple studies. Soy is an important component of the vegetarian diet in Taiwanese society to replace the protein source from red meat or fish. Such dietary habits could provide sufficient calories compared with a non-vegetarian diet (36). It has been confirmed that nutritional markers such as serum albumin and BMI are similar when a low-protein diet is applied. From the clinical evidence, the low-protein diet habit provided a protective role in lowering the overall mortality in the younger population from the NHANES III database (37). A recent meta-analysis from Naghshi et al. also provided evidence that all-cause mortality could be lessened by consuming a plant-based diet (38). From the aspect of mortality, the plant-based diet provides a benefit compared with a high-animal protein diet. In specific subgroups of CKD, a low-protein diet also provided clinical benefits. In pregnant CKD patients, the incidence of small for gestational age or extreme preterm babies was lowered when the patients used vegan-based protein restriction (16). From the aspect of homeostasis of calcium and phosphate in CKD, a vegetarian diet also played a conjunctive role in lowering the phosphate burden, while the body mass or fat might not be influenced (21). Beyond the consideration of religious beliefs, a vegetarian diet might be a safe dietary intervention when managing CKD.

There are still several limits in this study. This cohort study did not provide precise gradients of daily intake for all participants. This category was defined by the reply from the participants. We did not define the vegans by using the scales reflecting the daily food frequency, and the vegans or lacto-ovo vegans would not be digitalized. Further validated questionnaire such as 64-item food frequency questionnaire might be helpful to validate the accuracy of the self-report dietary habit. Therefore, the effect of calories and the proportion of protein could not be reflected directly. However, the study from Chiu et al. (21) demonstrated that the caloric content in vegetarian and non-vegetarian was similar for patients in Tzu-Chi medical system (1,705 vs 1,740 kcal, *p* = 0.11). The percentage of protein was 13 ± 1% and 12 ± 1%, respectively. The more precise measurement on the composition of diet in each individual might be needed in future study. Second, the study was a retrospective study, not a longitudinal study. We used the SEM model to validate the effect of vegetarian diets. The SEM model could express the interactions between different variables to predict the specific disease. From a previous study, the SEM model helped validate the efficacy of biomarkers for predicting CKD, such as Kim-1 (22, 39). In DM patients, the SEM model also played an important role in predicting the risk of CKD. From a study by Lee et al. hyperuricemia also contributed to the occurrence of DM nephropathy based on the SEM model (10, 40). However, such a model could not demonstrate the longitudinal variation in physiological or biochemical parameters, such as the change in blood pressure and decline in estimated glomerular filtration. Third, the study population was from a single institute with the foundation of the Buddhist religion. Forty-one percent of the participants of the cohort study were vegans or lacto-ovo vegetarians, which might be higher than that in other cohort studies (40, 41). Therefore, further studies with large populations and longitudinal follow-up might be needed. Finally, our database used the result for the self-paid health exams at the health checkup center. As the definition of American Diabetes association, the diagnosis of diabetes mellitus should be confirmed based on the random sugar, the fasting sugar and glycosylated hemoglobin (HbA1c) and Oral Glucose Tolerance Test (42). HbA1c was a convenient measurement for diagnosing DM. Our database measured HbA1c only, and therefore the diagnosis of diabetes mellitus could not be fulfilled according to ADA. However, the HbA1c might not detect DM with advanced CKD. However, the advanced CKD in our database was 1%, and the false-negative effect of HbA1c might not occurred. Besides, the effect of medication such as anti-diabetic or anti-hypertensive medications and the legacy of diabetes were not assessed in the study. The classification of diabetes mellitus, such as insulin deficient diabetes mellitus or mature onset of diabetes of the young could not be differentiated. The connection between the self-paid examination with the medical record in Tzu-Chi Medical system might provide more comprehensive aspects to understanding the different effect of diabetes mellitus.

In summary, the study investigated the role of lacto-ovo vegetarian and vegan diets in DM nephropathy. In our study, vegan and lacto-ovo vegetarian diets decreased CKD in DM patients. The protective effect of a vegan diet might be mediated by alleviating hyperuricemia.

## Data Availability Statement

The original contributions presented in the study are included in the article/supplementary material, further inquiries can be directed to the corresponding author/s.

## Ethics Statement

The study was approved by the Institutional Board of Taipei Tzu-Chi Hospital based on the Declaration of Helsinki (06-XD12-033). Further written informed consent were waived in this retrospective study by the Ethical Committee of Taipei Tzu-Chi Hospital.

## Author Contributions

Y-CH drafted the manuscript. J-SL executed statistical analysis of the dataset. S-YH and H-WL provided the data base. W-HT, H-FH, and K-LK designed the study and revised the manuscript. All authors contributed to the article and approved the submitted version.

## Conflict of Interest

The authors declare that the research was conducted in the absence of any commercial or financial relationships that could be construed as a potential conflict of interest.

## Publisher’s Note

All claims expressed in this article are solely those of the authors and do not necessarily represent those of their affiliated organizations, or those of the publisher, the editors and the reviewers. Any product that may be evaluated in this article, or claim that may be made by its manufacturer, is not guaranteed or endorsed by the publisher.
